# A Nationwide Cross-Sectional Study of Self-Reported Adherence and Factors Associated with Analgesic Treatment in People with Chronic Pain

**DOI:** 10.3390/jcm9113666

**Published:** 2020-11-14

**Authors:** Patricia Ortega-Jiménez, Helena De Sola, Alejandro Salazar, María Dueñas, Leticia Del Reguero, Inmaculada Failde

**Affiliations:** 1 The Observatory of Pain, University of Cádiz, 11009 Cádiz, Spain; patricia.ortega@uca.es (P.O.-J.); alejandro.salazar@uca.es (A.S.); maria.duenasro@uca.es (M.D.); leticiadelreguero@gmail.com (L.D.R.); inmaculada.failde@uca.es (I.F.); 2 Department of Statistics and Operational Research, University of Cádiz, 11510 Puerto Real, Spain; 3 Preventive Medicine and Public Health Area, University of Cádiz, 11009 Cádiz, Spain; 4 Biomedical Research and Innovation Institute of Cádiz (INiBICA), 11009 Cádiz, Spain

**Keywords:** adherence, chronic pain, analgesic treatment, cross-sectional study

## Abstract

This study aims to shed light on the frequency and associated factors of self-reported adherence to analgesic treatment among chronic pain (CP) patients in the Spanish population. A nationwide cross-sectional study was performed of 1066 Spanish adults, of whom 251 suffered from CP and 168 had been prescribed analgesic treatment. Adherence was assessed using a self-reported direct questionnaire and related factors were collected. Descriptive and bivariate analyses were conducted. Among the 23.5% (95% CI: 21.0–26.2%) of the sample with CP, 66.9% (95% CI: 60.7–72.7%) were taking analgesic treatment prescribed by a doctor, and 81.0% (95% CI: 74.2–86.6%) said they took the treatment as the doctor indicated. However, 17.6% forgot to take the medication, 11% overused them when in great pain, 46.3% stopped the treatment when feeling better and 33.3% when feeling worse, and 7.3% stopped taking them for financial reasons. Higher intensity of pain, polymedication, administration route (injection/patches) and some patient-related factors were associated with self-perceived adherence to treatment. Most Spanish people with CP consider that they are adherent to their analgesic treatment. However, their behavior presents contradictions. It would be advisable for professionals to inform patients about appropriate behavior regarding their therapy recommendations, and to explore potential factors related to non-adherence. This could contribute to improving pain control.

## 1. Introduction

Chronic pain (CP) is a major public health problem, recognized as a chronic disease entity [1], that affects between 10% and 30% of the adult population in Europe [2,3] and around 17% of the Spanish population [4]. The pain experience also has great impact on the physical and mental health of the patients, and on their working, family and social lives, leading to a heavy burden on the healthcare system [5,6,7].

Despite its relevance and the availability of treatments for pain relief, the management of CP needs to be improved [8]. In Spain, as in other countries such as Portugal or Norway [9,10,11], pain control is far from optimal, as 35% of CP patients taking treatment still refer to severe pain [6,8]. Several barriers have been related to pain control, associated with a lack of time for patient care, poor coordination between units involved in the management of these patients, poor communication between healthcare professionals or limited availability of specialized services [8]. 

Despite these barriers, pharmacological therapy is the most common way of dealing with pain, and adherence to the treatment is therefore considered an important factor in achieving this aim [12,13]. Adherence to treatments among people with CP has been shown to be inconsistent [13], ranging from 8–62%. This variability has been associated not only with the different terminology used to define the adherence concept, but also with the methods used for its assessment, making it difficult to compare results between studies and apply them in clinical practice [14].

Adherence to medication can be affected by a variety of demographic, clinical or socioeconomic factors [15]. The World Health Organization (WHO) has classified these factors into five dimensions: those related to socioeconomic conditions, since a low socioeconomic status may put patients in the position of having to choose between priorities; those associated with the pathology, such as the severity of the disease (physical, psychological) or patients’ perception of risk; those related to the treatments (complexity of the treatment, duration of treatment, beneficial effects, side effects, etc.); characteristics of the patients (forgetfulness, beliefs, perceptions, etc.); and those related to the health system and its professionals, given that a good patient–provider relationship may improve adherence, and many factors such as lack of knowledge or lack of feedback on performance could have a negative effect [16]. The analysis and identification of obstacles related to these factors could contribute to improving both adherence and patients’ health outcomes. 

Additionally, it has been shown [17] that a lack of adherence among patients may be either intentional or unintentional. Non-adherence is considered unintentional when a patient is unable to adhere with medical recommendations because of lack of capacity or resources, such as low health literacy. Meanwhile, intentional non-adherence refers to an active decision of the patient to not adhere to their physician’s instructions, which can be influenced, among other matters, by individual perceptions about the disease [17]. 

In Spain, several studies have investigated the adherence to analgesic therapy in the CP population [2,18] but, to the best of our knowledge, none have analyzed the adherence to medication in the context of the five dimensions defined by the WHO. Thus, we carried out this study, the main objective of which was to ascertain the frequency of self-reported adherence to analgesic treatment among the general Spanish population with CP, and to analyze the socioeconomic, health care, treatment, pathology and patient characteristics related to adherence to medication. 

## 2. Materials and Methods

### 2.1. Participants and Recruitment

A nationwide epidemiological cross-sectional study was carried out on a representative sample of the general Spanish population, with the target population including individuals in households with a landline telephone. The target population included people ≥18 years old living at the household selected who agreed to participate in the study and were able to complete the questionnaire. The exclusion criteria were: age <18 years old, a nationality other than Spanish, not living at the selected address, no landline telephone in the house, cancer pain or any inability to respond to the questionnaire.

### 2.2. Sampling Methods

A multistage stratified sampling method was carried out in three phases. In the first, the Spanish territory was divided into 8 strata based on geographical and historical boundaries and according to an aging criterion based on the ratio between people over 65 and under 15 years old, as seen in [19]. For each stratum, a number of municipalities were randomly selected proportional to the stratum weight in the total population, considering the Spanish rural (<10,000 inhabitants)/urban (>10,000) ratio of 25:75. A total of 20 municipalities were chosen. This division was considered as it has been shown that “aging” and “geographic zone” are factors that influence chronic pain prevalence [20,21,22,23,24]. 

In the second phase, sampling units (telephone numbers) from each town were selected from the list of telephone numbers included in the Infobel España Office v.7.1 directory^®^ (Kapitol S.A, Brussels, Belgium) by simple random sampling, until the quota of each stratum was fulfilled. This directory includes the telephone numbers of 90% of all Spanish households [25] with a landline telephone and, considering that 80.6% of Spanish households have one [26], we had access to 72.5% of the eligible Spanish population. 

In the third phase, the number of subjects was divided into 6 strata according to the sex and age distribution of the population: male aged 18–44; female aged 18–44; male aged 45–64; female aged 45–64; male aged ≥65; and female aged ≥65. In each of the municipalities, quotas were established, taking into account sex and age based on data on the Spanish population [27]. The subjects interviewed from the households were randomly selected according to the previously established sex and age quotas, and they were distributed in proportion to the size of each previously selected municipality. Within the household, the criteria were to select the first person who replied, as long as the quota of their stratum was not completed. In the event that the quota was completed, the selection of the other person of the household was established in the following order: men 18–44 years old; women 18–44 years old; men over 65 years old; women over 65 years old; men 45–64 years old; women 45–65 years old. The order was established according to the difficulty in filling each quota.

### 2.3. Sample Size

The sample size was calculated, bearing in mind the results obtained from a pilot study previously carried out by the authors, where the prevalence of CP was around 22%, and 67% of the subjects with CP were under analgesic treatment, and based on previous reports of adherence prevalence in Spain of 45.34% [2]. 

Thus, considering a confidence level of 95% and a precision of 8%, the required sample size was established at 1014 subjects. The sample size was finally adjusted upwards to 1066 subjects, as a preventive measure for missing data.

### 2.4. Procedure and Instruments

From February to June 2017, data were collected between 4 p.m. and 8 p.m. from Monday to Friday using a computer-assisted telephone interview, and the Skype^®^ (Skype Communications SARL, Luxembourg) and SurveyMonkey^®^ platforms (SurveyMonkey Europe UC, Dublin, Ireland). Previously, the interviewers received a 10 h workshop in which they were trained in the purpose of the study, the working protocol and the use of the SurveyMonkey^®^ platform. Furthermore, a pilot test was conducted during the previous week to analyze the comprehensibility and acceptability of the questionnaire and to detect and correct any potential problems related to the questions and the data collection procedure. 

The collection of data was coordinated and supervised on a daily basis by a member of the research team, addressing any problems that had arisen. 

### 2.5. Ethical Statement

Participation in the study was voluntary. All subjects included in the study were informed of the purpose and design of the research, and reassured that their identities and answers to the questionnaire were confidential. The subjects were informed of their right to withdraw from the interview at any moment. All subjects gave their oral consent for inclusion before they participated in the study. The study was conducted in accordance with the Helsinki Declaration and following standard working procedures. The study was approved by the Clinical Research Ethics Committee of Cádiz (Spain) (ASC-DC-2018), which declares and certifies that this project is viable, has sufficient methodological rigor and a correct assessment of the economic cost, and complies with the necessary requirements regarding the suitability of the protocol in terms of the aims of the study.

### 2.6. Survey Structure 

The survey was structured in 5 blocks, taking into account the factors defined by The World Health Organization (WHO) [16]. The first block was designed to obtain sociodemographic information such as age, sex, educational level, employment status or financial difficulties, assessed by asking about the difficulties found making ends meet each month. This block also contained a question used to identify subjects with CP. Accordingly, a subject was considered to suffer from CP if they had experienced pain (at least 4 days a week) over the last 3 months, in accordance with the International Association for the study of Pain (IASP) definition [28].

In the second block of the survey, dealing with CP, the subjects were asked about condition-related factors, such as diagnoses, duration and pain intensity, and about the presence of other physical comorbidities. Pain intensity was measured on a numerical rating scale (0–10), where higher scores represented more intense pain, and grouped into three categories to facilitate the analysis: “mild” (1–3), “moderate” (4–6), “severe” (7–10) [29,30]. The presence of depression and anxiety was also assessed, using the questions: “Have you suffered from depression (or anxiety) during the previous 12 months?” and “Has your medical doctor confirmed the diagnosis?” [19]. A patient was considered to have diagnosed depression (or anxiety) if both answers were affirmative.

The third block started with a screening question to identify those people with CP taking analgesic treatment prescribed by a doctor. From this question, and until the end of the interview, only people with CP that had been prescribed analgesic treatment were considered. For the purposes of this study, adherence to analgesic medication was measured in this block using the self-reported direct question (SRDQ) “Do you take your prescription as the doctor indicated?” When the answer to this question was negative, two questions were asked “Do you take more?” and “Do you take less?” In this block, information was also collected about factors related to the treatment, such as duration of the treatment, type and route of the treatment administration and about expectations of improvement, opinions of the pain treatment, addiction and side effects related to analgesic therapy.

The fourth block of the questionnaire included patient-related factors, such as forgetfulness, “Do you ever forget to take your pain medicine?”, questions related with the behavior of the patient “If you ever feel more pain, do you take more medicine than prescribed?”, “Have you ever stopped taking medication due to its price or skipped doses so drugs last longer?” and the perception of their health status. 

Finally, with the fifth block we explored healthcare professional- and family-related factors such as opinions of the respondent about the information and attention received from their doctor and family. 

### 2.7. Statistical Analysis

A descriptive analysis was performed, using measures of frequency (absolute and relative), central tendency (mean) and dispersion (standard deviation). To check the normality in the distribution of the quantitative variables, the Kolmogorov–Smirnov test was used. To analyze the associations between variables, χ^2^ tests were used for the qualitative variables, and the Mann–Whitney U test for quantitative variables. The threshold for significance was set at *p* = 0.05.

These analyses were all carried out with the IBM SPSS Statistics 22^®^ (IBM Corporation, Armonk, NY, USA) statistical package. 

## 3. Results

### 3.1. Characteristics of the Sample

**Figure 1:**
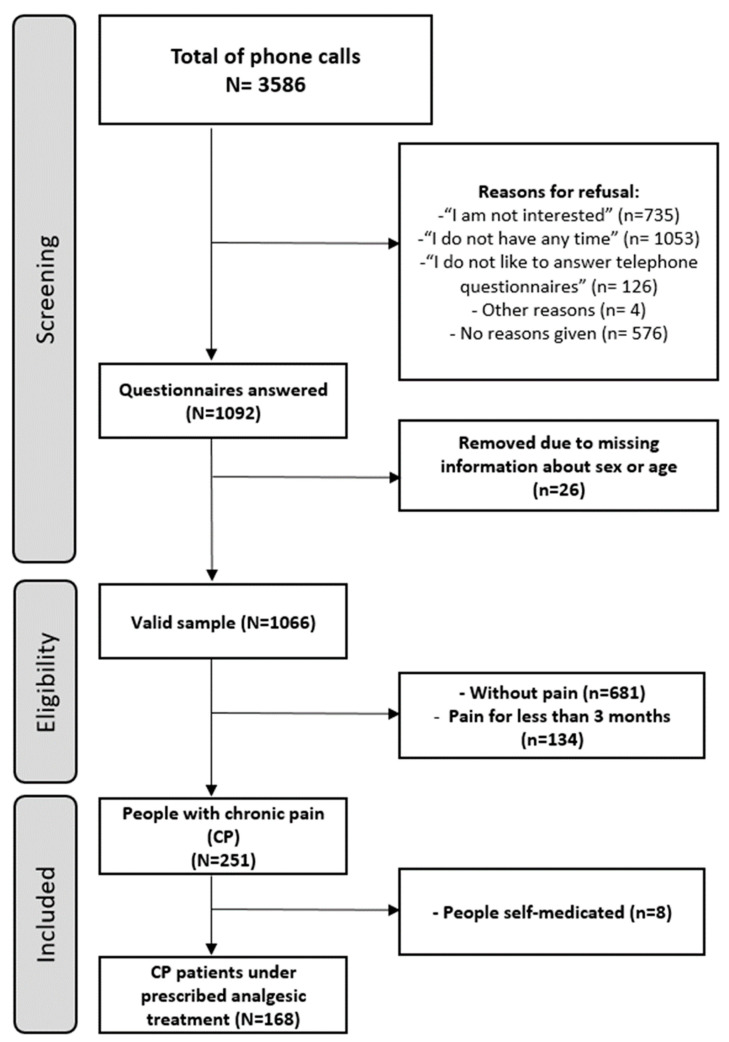
Flowchart of the sample.

Of the 3586 people contacted, 1092 were interviewed, and a response rate of 30.5% was achieved. The main reason for refusing to participate was “I do not have time” (42.2%), followed by “I am not interested in the topic” (29.2%). In 23.1% of the cases, no reason for refusal was given (Figure 1). Additionally, 26 subjects were excluded because of missing information about their sex or age. Therefore, the final number of subjects included in the study was 1066. The characteristics of the total sample are shown in Table 1.

**Figure 2:**
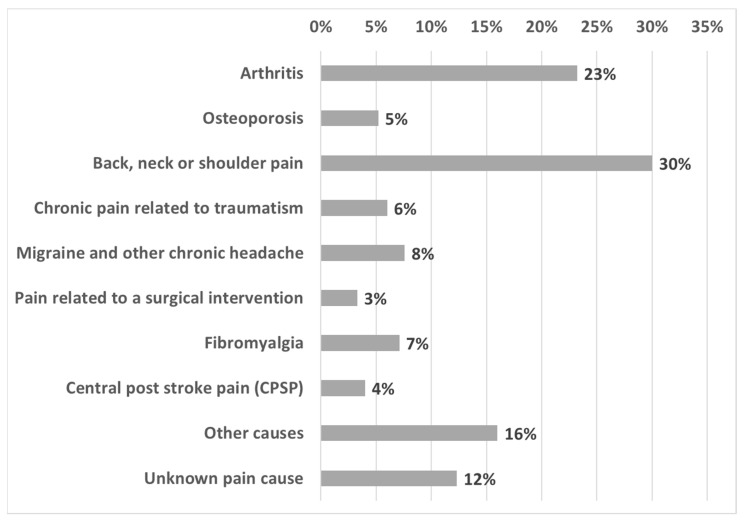
Pain diagnosis (*n* = 251).

Of the participants, 251 subjects (23.5%; 95% CI: 21.0–26.2%) referred to CP; 68.9% were women and the mean age was 55.9 (SD = 15.5). Pain intensity ≥7 was reported by 54.9%, and the mean duration of pain was nearly nine years (105.92 months, SD = 125.45; median = 60 months), back, neck or shoulder pain being the most frequent cause (30%), followed by arthritis (23%) (Figure 2). 

Among the people with CP, 66.9% (95% CI: 60.7–72.7%) were taking analgesic treatment prescribed by a doctor (*n* = 168), and 3.2% (*n* = 8) were self-medicated. In the group taking treatment, 76.2% were women, the mean age was 57.1 (SD = 15.6), only 34.1% were working and 53.4% had financial difficulties (Table 1). The average pain intensity of the people with CP taking analgesic treatment was over 7 points, and the duration of the pain was more than 10 years.

### 3.2. Comparison of the Factors Related to Self-Reported Adherence among People with CP Taking Treatment

The findings of the study show that 81% (95% CI: 74.2–86.6%) of the sample claimed to take the treatment as the doctor indicated (self-reported adherence), and only 19% self-reported not doing so. 

In the comparison between both groups, we found that the sociodemographic and economic factors were similar. However, in the self-reported adherent group, there were more subjects that were inactive, retired or housewives/husbands than in the non-adherent group (Table 2).

Regarding the condition-related factors, we observed that people who self-reported being adherent had a higher intensity of pain, and the main causes of pain were migraine and osteoporosis. Nevertheless, the duration of pain and the presence of comorbidities such as anxiety or depression were similar in both groups (Table 2).

With regard to the therapy-related factors, the self-reporting adherence group reported more frequently taking more than one medication (81.7% vs. 57.7%), taking benzodiazepines and using more drugs parenterally or in patches than the non-adherent group. No differences between the two groups were observed in the opinion on side effects, the addiction associated with pain relief medications, the duration of the treatments or the expectations regarding them. (Table 2).

Related to patient factors, it is noteworthy that a high percentage of the patients self-reporting non-adherence stated that they forgot to take their medication (34.4% vs. 17.6%); stopped the treatment when they felt better (83.9% vs. 46.3%) and when they felt worse (45.2% vs. 33.3%); forgot to take the medication at the hours indicated (37.5% vs. 25.7%); and took more medication when they felt worse (18.8% vs. 11%). In the self-reporting adherence group, 7.3% referred to stopping taking the medication due to price, while 3.1% in the non-adherence group did so (Table 2).

Finally, the study also showed that 91.1% of the self-reported adherence group considered the attention received by the doctor to be acceptable, good or very good, while 81.3% of the non-adherent group felt the same; and 93.4% considered the information provided by the health professional to be acceptable, good or very good (versus 87.5%), although the latter was not significant. The majority of the sample in both groups were satisfied or very satisfied with their family support (70% and 75%, respectively) (Table 2).

## 4. Discussion

This study analyzes the self-reported adherence to analgesic therapy in people with CP in Spain, and compares the outcome according to the dimensions established by the WHO. The study showed that 81.0% of the people with CP self-reported adherence to their doctor’s recommendations. This data are similar to the findings in Portugal [31], where non-adherence was identified in 22.8% of the people with chronic pain conditions and different to the data from Belgium, where 48% of the patients were non-adherent to the treatment [32]. However, when we explored our findings more deeply, we found that the majority of them did not behave according to their physician’s recommendations. 

A possible explanation for this is that the information given to patients by their healthcare professionals is often misunderstood, carried out incorrectly, forgotten or even completely ignored. In line with these results are those published by the WHO in 2013 [33], showing than more than half of the Spanish population has inadequate or problematic health literacy levels, which is associated with poor adherence to medication [33].

Another potential explanation contributing to the high rates of self-reported adherence observed could be social desirability patterns, whereby patients feel the need to be a “good patient” so as not to be seen as negligent [34,35]. 

In our study, nearly 20% of the participants that self-reported adherence to therapy stated that they forgot to take the medication. However, to date, forgetfulness has not been considered a determinant for medication non-adherence in patients with CP, which may be related with the persistent presence of the unpleasant symptom [36].

The findings also show that the self-reported adherent group presented worse conditions, such as a higher intensity of the pain compared to the non-adherent group. Depending on the pathology and the intensity of pain, an individual may receive more than one drug during the course of the illness and through different administration routes, which could have an effect on adherence [15]. As shown in our study, the subjects reporting polymedication and using patches or injections more frequently self-reported adherence. This is comprehensible because these situations require more attention, more information and control by health professionals.

It is obvious that the intention to perform an action constitutes an immediate precursor to the behavior itself [37]. If patients hold beliefs that are incongruent with their doctor’s prescription, or if their social group holds different points of view about their illness and treatment, patients may intentionally develop a pattern of behavior that corresponds to lack of agreement with the recommendations [38]. In fact, other studies showed that the prevalence of intentional non-adherence is higher than unintentional non-adherence [32,39]. This was reaffirmed in our study, where we found that, among patients that had self-perceived adherence, the main reason for non-adherence was intentionally stopping taking the medication when the patient felt worse or better than expected. 

One important problem related to adherence is that there is no suitable standard for its evaluation in patients with CP [10,13]. This leads to uncertainty about the impact on clinical outcomes [14,15]. Despite this limitation, several authors report a negative correlation between adherence with analgesic therapy and pain intensity [18,39], and they state that adherence reduces the costs associated with health care [39,40]. Other authors have reported that non-adherence undermines treatment benefits and is associated with poorer prognosis [40]. Therefore, ensuring agreement with the treatment plan is an essential part of patient care and an important objective in the optimal management of pain. 

The prevalence of CP in our study was 23.5% (95% CI: 21.0–26.2%). This is a bit higher than in another study performed in the Spanish population [4], where the prevalence was 17%. This could be related to the fact that this study was performed in 2011, nearly 10 years ago, and taking into account that the prevalence of multiple chronic medical conditions is increasing worldwide [41]. Worryingly, these problems related to CP are expected to increase [42] because of the increase in life expectancy and aging trends in the workforce, resulting in more painful conditions, especially musculoskeletal ones [43]. 

Finally, some methodological limitations need to be considered in this study. The first arises with the use of a dichotomous answer to summarize self-reported adherence to therapeutic recommendations. However, adherence is widely measured as a dichotomous variable [13] in the absence of a gold standard to assess it. Moreover, we used other complementary questions to study this concept based on the dimensions established by the WHO. In the study, we analyzed patients’ perceptions of adherence, and we had the opportunity to contrast their answers with other items, such as stopping taking the treatment when feeling better or due to its price. This gave us a wider view of the behavior of CP sufferers, and allowed us to detect the misconceptions that led people to claim to be adherent when they were not necessarily so. 

The second limitation to consider is that, since it was a population-based study and the interviews were conducted by telephone, objective methods for measuring adherence were not feasible. Furthermore, ten interviewers took part in the survey. Therefore, although a strict protocol was established to minimize discrepancies, a slight bias may have existed due to the attitude of each interviewer. Another limitation is that information about how the patients took their medication was not collected. Therefore, we could not identify if they took it on a pro re nata (prn) or “as required” basis for pain, meaning the decision of when to administer the medication is left to the patient, as circumstances required, under a previously prescribed medication regimen. It should be noted that the response rate in this study (30.5%) was low. However, the reasons for those not responding were collected, and none of them are known to lead to bias. Furthermore, according to the literature, it is not unusual to find similar rates in these types of studies [31,44]. 

## 5. Conclusions

Most Spanish people with CP consider that they are adherent to their analgesic treatment. However, their behavior presents contradictions. High intensity of pain, polymedication, the administration route of the treatment (injection and patches) and some patient-related factors were associated with self-perceived adherence to treatment. 

It would be advisable for professionals to inform patients about appropriate behavior regarding their therapy recommendations, and to explore potential factors related to non-adherence. This could contribute to improving pain control. 

## Figures and Tables

**Table 1:** Characteristics of the sample.

Variables	Categories	Whole Sample (*n* = 1066) *n* (%)	People with Chronic Pain (CP) (*n* = 251) *n* (%)	People with CP Taking Analgesic Treatment (*n* = 168) *n* (%)
SOCIODEMOGRAPHIC DATA				
Age	18–44 45–64 65 or more	424 (39.8) 385 (36.1) 257 (24.1)	72 (28.7) 105 (41.8) 74 (29.5)	45 (26.8) 68 (40.5) 55 (32.7)
Age	Mean (SD)	51.6(16.5)	55.9 (15.5)	57.1 (15.6)
Sex	Men Women	456 (42.8) 610 (57.2)	78 (31.1) 173 (68.9)	40 (23.8) 128 (76.2)
Education level	No education received Primary school Secondary school Vocational training University degree	83 (7.8) 215 (20.3) 252 (23.8) 162 (15.3) 348 (32.8)	31 (12.4) 73 (29.1) 54 (21.5) 42 (16.7) 51 (20.3)	21 (12.5) 49 (29.2) 37 (22) 29 (17.3) 32 (19)
Employment status	Unemployed Student Housewife/househusband Working Retired Partial disability Total disability	147 (13.8) 54 (5.1) 125 (11.8) 484 (45.6) 240 (22.6) 6 (0.6) 6 (0.6)	41 (16.4) 4 (1.6) 43 (17.2) 92 (36.8) 60 (24) 5 (2) 5 (2)	29 (17.4) 3 (1.8) 34 (20.4) 57 (34.1) 39 (23.4) 2 (1.2) 3 (1.8)
Financial difficulties	With great difficulty With difficulty With some difficulty With some ease With ease With great ease	65 (6.2) 137 (13) 201 (19.1) 272 (25.9) 339 (32.3) 37 (3.5)	30 (12) 45 (18.1) 60 (24.1) 59 (23.7) 53 (21.3) 2 (0.8)	20 (12) 29 (17.4) 40 (24) 45 (26.9) 32 (19.2) 1 (0.6)

**Table 2:** Comparison of the factors related to self-reported adherence among people with chronic pain taking treatment.

Variables		Categories	Do you Take your Prescription as Indicated by the Doctor?		*p*
			NO (*n* = 32; 19%)	YES (*n* = 136; 81%)	
**Social and economic factors**					
Age		18–44 45–64 65 or more	8 (25%) 16 (50%) 8 (25%)	37 (27.2%) 52 (38.2%) 47 (34.6%)	0.44 ^a^
Sex		Men Women	8 (25 %) 24 (75%)	32 (23.5%) 104 (76.5%)	0.86 ^a^
Level of education		No education received Primary school Secondary school or vocational training University studies	1 (3.1%) 10 (31.1%) 14 (43.8%) 7 (21.9%)	20 (14.7%) 39 (28.7%) 52 (38.2%) 25 (18.4%)	0.36 ^a^
Employment status		Inactive Active Housewife/househusband Retired	6 (18.8%) 17 (53.1%) 4 (12.5%) 5 (15.6%)	28 (20.7%) 43 (31.9%) 30 (22.2%) 34 (25.2%)	0.14 ^a^
How difficult is it for you to make ends meet?		With great difficulty With some difficulty With ease	9 (29%) 7 (22.6%) 15(48.4%)	40 (29.4%) 33 (24.3%) 63 (46.3%)	0.97 ^a^
**Condition-related factors**					
How long have you been suffering from this pain? (months)		Mean (SD)	95.97 (98.21) (*n* = 31)	118.14 (131.73) (*n* = 135)	0.89 ^c^
Have you been diagnosed with depression during the previous 12 months?		No Yes	28 (87.5%) 4 (12.5%)	109 (80.1%) 27 (19.9%)	0.33 ^a^
Have you been diagnosed with anxiety during the previous 12 months?		No Yes	24 (75%) 8 (25%)	99 (72.8%) 37 (27.2%)	0.80 ^a^
On a scale of 0 to 10, how would you rate the intensity of your chronic pain during the last week?		Mean (SD)	6.47 (1.525) (*n* = 30)	7.29 (1.67) (*n* = 127)	0.015 ^c^
Osteoporosis is the main diagnosed cause of the pain		No Yes	32 (100%) 0	126 (93.3%) 9 (6.7%)	0.047 ^b^
Migraine is the main diagnosed cause of the pain		No Yes	32 (100) 0	125 (92.6%) 10 (7.4%)	0.036 ^b^
**Therapy-related factors**					
How long have you been taking the pain medication? (months)		Mean (SD)	79.52 (93.75) (*n* = 29)	89.91(117) (*n* = 124)	0.874 ^c^
Could you tell me which medication you have been prescribed for the pain?	NSAIDs (M01A)	No Yes	10 (37.0%) 17 (63.0%)	58 (48.3%) 62 (51.7%)	0.287 ^a^
	Antipyretic analgesics (N02B)	No Yes	14 (51.9%) 13 (48.1%)	72 (60.0%) 48 (40.0%)	0.44 ^a^
	Opioids (N02A)	No Yes	24 (88.9%) 3 (11.1%)	89 (74.2%) 31 (25.8%)	0.10 ^a^
	Antidepressant (N06A)	No Yes	26 (96.3%) 1 (3.7%)	114 (95.0%) 6 (5.0%)	0.77 ^b^
	Antiepileptic (N03A)	No Yes	26 (96.3%) 1 (3.7%)	111 (92.5%) 9 (7.5%)	0.45 ^b^
	Others (benzodiazepines, cortisone, etc.)	No Yes	27 (100%) 0 (0%)	98 (81.7%) 22 (18.3%)	0.002 ^b^
How many medications are you taking in total at the moment (not just for the pain)?		One Two or three Four or five More than five	13 (43.3%) 12 (40%) 4 (13.3%) 1 (3.3%)	26 (19.3%) 59 (43.7%) 23 (17%) 27 (20%)	0.016 ^a^
How do you take the pain medication?	Orally	No Yes	0 (0%) 30 (100%)	6 (4.4%) 130 (95.6%)	0.118 ^b^
	Injections	No Yes	29 (96.7%) 1 (3.3%)	112 (82.4%) 24 (17.6%)	0.023 ^b^
	Patches	No Yes	30 (100%) 0 (0%)	125 (91.9%) 11 (8.1%)	0.033 ^b^
Has your pain improved as you expected with the treatment?		Less than expected As expected More than expected	15 (48.4%) 12 (38.7%) 4 (12.9%)	58 (42.6%) 55 (40.4%) 23 (16.9%)	0.79 ^a^
Patients that take medication for pain can easily become addicted to it		Disagree Somewhat agree Agree	10 (32.3%) 4 (13.9%) 17 (54.8%)	44 (33.3%) 39 (29.5%) 49 (37.1%)	0.101 ^a^
Medication used to treat pain leads to side effects		Disagree Somewhat agree Agree	5 (16.1%) 6 (19.4%) 20 (64.5%)	32 (23.7%) 36 (26.7%) 67 (49.6%)	0.32 ^a^
**Patient-related factors**					
Do you ever forget to take your pain medicine?		No Yes	21 (65.6%) 11 (34.4%)	112 (82.4%) 24 (17.6%)	0.036 ^a^
Do you ever forget to take medications at the hours indicated?		No Yes	20 (62.5%) 12 (37.5%)	101 (74.3%) 35 (25.7%)	0.18 ^a^
If you ever feel better, do you stop taking the treatment?		No Yes	5 (16.1%) 26 (83.9%)	73 (53.7%) 63 (46.3%)	0.000 ^a^
If you ever feel bad, do you stop taking the treatment?		No Yes	17 (54.8%) 14 (45.2)	90 (66.7%) 45 (33.3%)	0.21 ^a^
If you ever feel more pain, do you take more medicine than prescribed?		No Yes	26 (81.3%) 6 (18.8%)	121 (89.0%) 15 (11.0%)	0.26 ^b^
Have you stopped taking the medication due to its price?		No Yes	31 (96.9%) 1 (3.1%)	126 (92.6%) 10 (7.4%)	0.35 ^b^
Have you skipped a dose so that the medication lasts longer?		No Yes	29 (90.6%) 3 (9.4%)	129 (94.9%) 7 (5.1%)	0.39 ^b^
In general, you would say your health is…		Excellent or very good Good Normal Bad	0 (0 %) 17 (53.1%) 12 (37.5%) 3 (9.4%)	8 (5.9%) 58 (42.6%) 51 (37.5%) 19 (14.0%)	0.22 ^b^
**Healthcare professional- and family-related factors**					
The information that your doctor has given you about your treatment for pain was…		Very good or good Acceptable Bad or very bad	16 (50%) 12 (37.5%) 4 (12.5%)	84 (61.8%) 43 (31.6%) 9 (6.6%)	0.36 ^a^
The attention provided by your doctor regarding your pain was…		Very good or good Acceptable Bad or very bad	16 (50%) 10 (31.3%) 6 (18.8%)	103 (75.7%) 21 (15.4%) 12 (8.8%)	0.016 ^a^
Are you satisfied with the help received from your family for your pain?		Unsatisfied Satisfied Very satisfied	8 (25%) 15 (46.9%) 9 (28.1%)	41 (30.1%) 66 (48.5%) 29 (21.3%)	0.68 ^a^
Do you have a relative that reminds you to take your medication?		No Yes	25 (78.1%) 7 (21.9%)	103 (75.7%) 33 (24.3%)	0.77 ^a^

^a^ Pearson Chi-square; ^b^ Likelihood ratio; ^c^ Mann–Whitney U test.
